# Why noise is useful in functional and neural mechanisms of interval timing?

**DOI:** 10.1186/1471-2202-14-84

**Published:** 2013-08-07

**Authors:** Sorinel A Oprisan, Catalin V Buhusi

**Affiliations:** 1 Department of Physics and Astronomy, College of Charleston, Charleston, SC, USA; 2 Department of Psychology, Utah State University, Logan, UT, USA

## Abstract

**Background:**

The ability to estimate durations in the seconds-to-minutes range - interval timing - is essential for survival, adaptation and its impairment leads to severe cognitive and/or motor dysfunctions. The response rate near a memorized duration has a Gaussian shape centered on the to-be-timed interval (criterion time). The width of the Gaussian-like distribution of responses increases linearly with the criterion time, i.e., interval timing obeys the scalar property.

**Results:**

We presented analytical and numerical results based on the striatal beat frequency (SBF) model showing that parameter variability (noise) mimics behavioral data. A key functional block of the SBF model is the set of oscillators that provide the time base for the entire timing network. The implementation of the oscillators block as simplified phase (cosine) oscillators has the additional advantage that is analytically tractable. We also checked numerically that the scalar property emerges in the presence of memory variability by using biophysically realistic Morris-Lecar oscillators. First, we predicted analytically and tested numerically that in a noise-free SBF model the output function could be approximated by a Gaussian. However, in a noise-free SBF model the width of the Gaussian envelope is independent of the criterion time, which violates the scalar property. We showed analytically and verified numerically that small fluctuations of the memorized criterion time leads to scalar property of interval timing.

**Conclusions:**

Noise is ubiquitous in the form of small fluctuations of intrinsic frequencies of the neural oscillators, the errors in recording/retrieving stored information related to criterion time, fluctuation in neurotransmitters’ concentration, etc. Our model suggests that the biological noise plays an essential functional role in the SBF interval timing.

## Background

**Figure 1:**
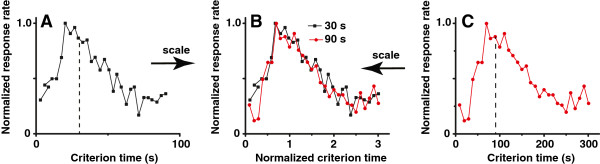
**Interval timing is precise and scalar.** Normalized mean lever-press response rate in peak-interval experiments with rats trained with a criterion time of 30s **(A)**, respectively, 90s (**C**; re-drawn from [13]). When normalized by the maximum response rate and by the criterion duration on the horizontal scale, response functions for the two criteria overlap **(B)**.

The capability of perceiving and using the passage of time in the seconds-to-minutes range (interval timing) is essential for survival and adaptation, and its impairment leads to severe cognitive and motor dysfunctions [1-3]. In most species, interval timing is both *accurate* and *time-scale invariant*, or simply scalar, in that the error in time estimation is proportional to the estimated duration [4-6]. When timing a 30s interval (Figure 1A), responses follow a quasi-Gaussian distribution around the 30s target duration. Furthermore, when timing a 90s interval (Figure 1C), responses also follow a quasi-Gaussian distribution around the 90s target duration. The scalar property is evident in that normalizing the response functions by the target duration and by the maximum response rate yields superimposition of response functions (Figure 1B). The time-scalar invariance property of interval timing is ubiquitous in many species from invertebrates such as bees [7], to many vertebrates, such as fish [8], birds [9], and mammals such as rats [10], mice [11] and humans [12].

Although the localization of brain regions involved in interval timing is not yet clear, some progress has been made. For example, both temporal production and temporal perception are strongly connected to striatum and its afferent projections from the substantia nigra pars compacta [14-16]. In addition, it was shown that the firing patterns of striatal neurons peak around a trained criterion time, a pattern consistent with substantial striatal involvement in interval timing [17]. Pharmacological data also suggest a strong basal ganglia involvement in interval timing. Administration of dopaminergic drugs both systemically [13,18-23] or directly into the anterior portion of the striatum [24] alters the speed of interval timing. Experiments showed a shift in the perceived time towards earlier times following systemic dopamine (DA) agonists administration (e.g., methamphetamine or cocaine) whereas systemic DA antagonists administration (e.g., haloperidol) shift the response times in the opposite direction (clock pattern). A possible physiological hypothesis is that DA causes internal clock(s) to run faster than normal, therefore, shifting the entire response of the animal earlier than the control [20,25,26].

Studies done in humans also support the hypothesis that striatum and its afferents are involved in interval timing [27-33]. Recent fMRI data showed that timing network could involve cortico-striatal loops, including premotor and supplementary motor areas, frontal operculum and dorsolateral prefrontal cortex, and temporal and parietal cortices, as well as the putamen [34]. Imaging studies in humans [28-30,32], lesion studies in humans [31,35-37] and rodents [14,15,22], and drug studies in rodents [17-20,22-24,38] all point towards a central role of the basal ganglia in interval timing. The anatomy of the basal ganglia suggests that information is proceeded through cortico-striato-thalamic loops. Severe deficiencies in reproducing temporal intervals were also found in Parkinson’s patients, therefore, further supporting the hypothesis of basal ganglia involvement in interval timing [36,39-41]. There are also lesions data suggesting that the timing network is much widely distributed. Lesions of the nucleus basalis magnocellularis, a cholinergic nucleus in the basal forebrain with projections to the frontal cortex, induced a progressive, proportional, delay in peak time response (memory pattern). This effect is believed to be related to altered temporal memories [42,43] due to change in acetylcholine (ACh) level. Lesions of the frontal cortex produce similar memory patterns [44], whereas lesions of the hippocampus or fimbria fornix, a basal forebrain cholinergic nucleus with projections to the hippocampus, result in memory effects translated into an advance of the peak time response [42-44]. These experimental findings undoubtedly support the hypothesis of a distributed interval timing neural network.

The connectionist model is among the first attempts to integrate a large collection of experimental findings into a coherent distributed network model of interval timing by Church and Broadbent [45,46]. They assumed that a set of neural oscillators, probably localized in the prefrontal cortex, determines the peak time using multiple-period discrimination algorithms. In their model, the current phases of oscillators (clock stage) are continually compared against the memorized phases at the reinforcement time (memory stage). The connectionist model successfully duplicated the scalar property and the response form of both peak-interval [46] and fixed-interval procedures [47]. The connectionist model also presents higher accuracy for intervals near the underlying oscillator period similar to experimental observations [48-50]. However, the connectionist model is limited to timing durations that do not exceed the longest period of the set of oscillators and requires a quite large coefficient of variation [51].

Another successful distributed network model, called the beat frequency model, uses beats between multiple oscillators to produce a much wider range of durations than the intrinsic periods of individual oscillators [13,21,52]. It is assumed that at the beginning of each trial all oscillators are reset and start in phase. At the reinforcement time, the oscillators are read out to determine whether they are spiking (“on” state) or are silent (“off” state). The small group of neurons that spike at the reinforcement time represents the neural code for that particular duration. A temporal prediction is made by a threshold-driven comparison between the number of strengthened neurons currently firing and the number of neurons that fired at the reinforcement time. Miall [52] conducted numerical simulations using beat frequency model and found a second peak halfway through the criterion duration similar to the “breakpoint” time observed in the peak-interval procedure [53]. In addition, the third highest peak corresponds to 2/3 of the way to the criterion in a manner similar to the breakpoint seen in fixed interval procedures [54].

In this study, we generalized previous results regarding the quasi-Gaussian shape and the scalar property using the SBF model [13,21,52]. Although it was long assumed that the behavioral response curve for peak procedure could be approximated by a Gaussian, here we actually proved theoretically and checked numerically that it is always a Gaussian. Furthermore, we showed that this fact is independent of the type of variability, or biological noise, present in the interval timing network.

We also showed that the scalar property is a universal feature of any SBF model regardless the type of model neurons used and the type of probability distribution functions (*pfd*) for parameters affected by biological noise. Variability in the SBF model could be ascribed to channel gating fluctuations [55,56], noisy synaptic transmission [57], and background network activity [58-60]. Single-cell recordings support the hypothesis that irregular firing in cortical interneurons is determined by the intrinsic stochastic properties (channel noise) [61-63] of individual neurons [64,65]. At the same time, fluctuations in the presynaptic currents that drive cortical spiking neurons have a significant contribution to the large variability of the interspike intervals [66,67]. For example, in spinal neurons, synaptic noise alone fully accounts for output variability [66]. In this paper, we are not concerned with the biophysical mechanisms that generated irregular firing of cortical oscillators. We rather investigate if assumed variability in SBF model’s parameters can produce *precise* and *time-scale invariant* interval timing.

Within the SBF paradigm we used a simple model of cortical oscillators, i.e., a cosine wave (phase) model (see [52] and references therein) and showed analytically that it (a) violates the scalar property in the absence of model’s parameters variability (noise), and (b) the output function is always Gaussian and obeys the scalar property regardless the *pdf* of assumed model’s variability. The above two analytical predictions were numerically confirmed both with the cosine wave model oscillators and with a more biophysically realistic, conductance-based, Morris-Lecar (ML) model neuron [68,69]. ML model neuron was developed for the giant muscle fiber of barnacles [68] by combining Hodgkin-Huxley [70] and FitzHugh-Nagumo [71,72] models into a voltage-gated calcium channel model with a delayed-rectifier potassium channel. Since then, ML model was successfully used for describing different types of cortical neurons. For example, White *et al*[73] performed voltage-clamp recordings from entorhinal cortical neurons of mice and calibrated a ML model neuron in which they replaced the calcium current by an instantaneously-activated persistent sodium current. By comparing the results of ML model neuron against experimental data, they found that “this reduction in the number of dependent variables does not alter significantly the behavior of the system.” For this reason, and because the ML model is considered a canonical prototype for widely encountered classes of both deterministic and stochastic neurons [74], we used ML model in our implementation of the SFB model.

## Methods

**Figure 2:**
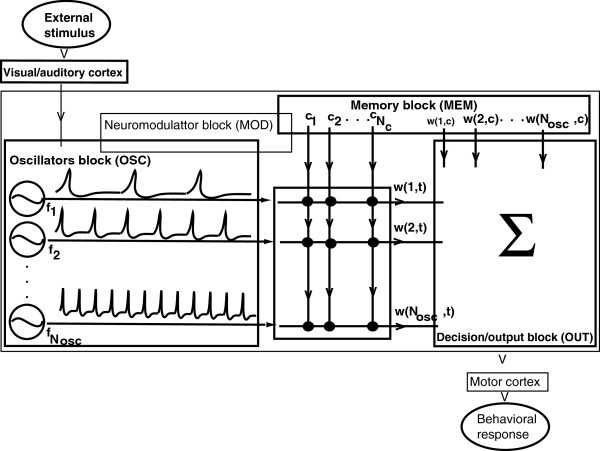
**Schematic representation of the functional blocks of the SBF model.** The oscillator block **OSC** contains *N*_*osc*_ neural oscillators that constitute the time base for the entire interval timing network. The memory block **MEM** stores the criterion time, *c*, and the “state” of the brain at the reinforcement time *w*(*k*,*c*), with *k*=1,…,*N*_*osc*_. The decision and output block **OUT** compares the current state of the oscillators in **OSC**, *w*(*k*,*t*), with the memorized reference state *w*(*k*,*c*) at reinforcement time and produces a smooth output proportional to the “closeness” of the two states. The neuromodulator block **MOD** mimics the global effect of DA and ACh neuromodulators.

We introduced a minimal block diagram that mimics the contributions of some of the neuroanatomical regions known to be involved in interval timing as identified in the Introduction. The schematic diagram includes the following blocks (see Figure 2). An oscillator block **(OSC)**, presumably mimicking the neural oscillators localized in the prefrontal cortex area [17]. A memory block **(MEM)**, presumably mimicking the activity associated with the nucleus basalis magnocellularis [42,43], frontal cortex [44], and/or hippocampus or fimbria fornix [42-44]. Its role is to stores the information about the state of the brain at the reinforcement time. A decision block **(OUT)**, presumably mimicking the striatal spiny neurons that by integrating a very large number of different inputs and responding selectively to particular reinforced patterns [75-77]. Finally, a neuromodulator block **(MOD)** that mimics the modulation of cortical and thalamic-induced activity of the striatal spiny neurons. The **MOD** block also modulates the threshold for coherent activity detection due to dopamine release from substantia niagra pars compacta [78].

### The oscillator block (OSC)

is composed of *N*_*osc*_ neural oscillators with frequencies distributed over a range (*f*_1_,*f*_2_) consistent with neurobiological observations [21,52,79-81]. The fixed firing frequencies of individual neural oscillators, *f*_*i*_, are equally spaced, i.e, *f*_*i*_=*f*_1_+*i*·*df* with frequency increments *df*=(*f*_2_−*f*_1_)/*N*_*osc*_. **OSC** provides the underlying time base for the interval timing network. In the presence of noise, e.g., ionic channel noise [55,56,61-65] or background neural activity from other cortical areas [57-60,66,67], a set *N*_*f*_ of *N*_*osc*_ frequencies, ${\tilde{f}}_{i}=f_{i}(1+x_{f})$, are generated from a random distribution around *f*_*i*_ with a frequency variability *x*_*f*_ that obeys a given probability density function *pdf*_*f*_. The output function is an average over all *N*_*f*_ distributions of frequencies.

### The memory block (MEM)

stores a criterion time value, *c*, memorized during the training process. Both storing and retrieving the criterion time to and from the long-term memory are affected by biological context (brain state, noise, etc.) Therefore, in the presence of noise, a set *N*_*c*_ of randomly distributing values $\tilde{c}=c(1+x_{c})$ are generated with the mean *c* and variability *x*_*c*_ according to a given probability density function *pdf*_*c*_. The output function averages over all *N*_*c*_ randomly distributed values of the criterion time *c*.

### The decision/output block (OUT)

relates the internal perception of time with external actions.

In order to implement the decision-making process ascribed to basal ganglia, we define a set of numbers (weights) that represent the state of each oscillator. The weight *w*(*k*,*c*) encodes the state of *k*^*th*^ neural oscillator from the **OSC** block at the reinforcement (criterion) time. Although it is not the only possibility, the “state” of the brain at the reinforcement time could be given, for example, by the phases or the amplitudes of all neural oscillators in **OSC**. The **OUT** block estimates the “closeness” between the current state of the brain represented by the running weights *w*(*k*,*t*) and the memorized weights at the reinforcement time *w*(*k*,*c*). Among many possible implementations of the “closeness”, we chose the projection of the running weights *w*(*k*,*t*) along the vector of reference weights *w*(*k*,*c*) (the dot product of vectors *w*(*k*,*c*) and *w*(*k*,*t*)).

### The neuromodulator block (MOD)

mimics the experimentally observed effects of neuromodulators on interval timing. The actual mechanism implemented in this SBF model directly changes the firing frequency of all neurons in the **OSC** block proportional to the level of neuromodulator. In this implementation of the SBF model, we used the **MOD** block as a “start gun” that resets the **OSC** block at the beginning of each trial such that all neural oscillators state in phase. Elsewhere [82-85], we showed that a more detailed implementation of the DA modulation in the SBF model correctly reproduces the *clock patterns*: immediate change in timing and gradual re-calibration under the drug, immediate re-bound in the opposite direction and gradual re-calibration upon discontinuing the drug, and scalar (proportional) effects as observed in experiments (see, for example, [23]). Similarly, we showed [82-85] that manipulations of ACh level that modulates the long-term memory lead to *memory patterns*: gradual change in timing on-drug, gradual re-calibration upon discontinuing the drug, and scalar (proportional) effects (see [23] for comparison with experiments).

## The SBF model with cosine oscillators

In order to gain insight into the functionality of the SBF block model, we initially assumed that the time base is provided by cosine (phase) oscillators. A phase oscillator is a mathematical abstraction obtained by reducing a complex and detailed mathematical model of a biological neuron to a single parameter - the firing phase measured with respect to an arbitrary reference [86-90]. The simplest possible oscillatory behavior is represented by cos(2*πft*), where the argument of cosine is called the phase of oscillation, *t* is the temporal variable, and *f* is the fixed firing frequency of the oscillator. Phase oscillators represent a significant class of neural oscillators and all complex neural oscillators can be reduced to a phase oscillator near bifurcation points [91].

In our implementation of the SBF model, the reference weights *w*(*k*,*c*), which represent the state of the brain at the reinforcement (criterion) time are normalized values of the state of neuron *k*:

(1) $$w(k,c)=\overset{N_{c}}{\underset{i=1}{\sum }}\cos(2\pi {\tilde{f}}_{k}{\tilde{c}}_{i})/\text{Norm},$$

where the sum is considered over all stored criteria ${\tilde{c}}_{i}$ that fluctuate around *c* due to memory noise. The normalization factor is the maximum value over all states $\text{Norm}=\text{Max}(\overset{N_{c}}{\underset{i=1}{\sum }}\cos(2\pi {\tilde{f}}_{k}{\tilde{c}}_{i}))\leq N_{c}$, in which case the reference weight is bounded −1<*w*(*k*,*c*)<1. We also tested positively defined weights given by:

(2) $$w(k,c)=\overset{N_{c}}{\underset{i=1}{\sum }}\frac{\cos(2\pi {\tilde{f}}_{k}{\tilde{c}}_{i})+1}{2\text{Norm}},$$

and found no significant difference in the properties of the output function.

In this implementation of the SBF model, **OUT** works as a phase detector, i.e., if the current vector of weights *w*(*k*,*t*) at the current phases (time) matches the reference weights vector *w*(*k*,*c*) at criterion time, *c*, then a strong response is delivered, otherwise the response is negligible. In order to generate a response, the **OUT** block computes the current weights *w*(*k*,*t*) for each oscillator according to (1), or (2), and projects them along the reference weights vector *w*(*k*,*c*):

(3) $$\text{output}(t)=\overset{N_{\text{osc}}}{\underset{k=1}{\sum }}w(k,c)w(k,t).$$

Based on (3), we computed the absolute value of the cosine of the angle between *w*(*k*,*t*) and *w*(*k*,*c*), which smoothly varies between unity, when the current state of the brain coincides with the one memorized at the reinforcement time, and zero, when there is no overlap between them. Other common and equally appealing choices in computational neuroscience, but a bit more expensive from a computational point of view, are sigmoidal functions or double exponentials, both of which are often used to mimic experimentally measured activation/inactivation curves with smooth transitions between “off” (zero) and “on” (unity) states.

## Results

### Cosine oscillators with no variability

We gained significant insight into the dynamics of SBF model by assuming no noise (variability) in any of the model’s parameters. According to (1), the state of the **OSC** bock at the reinforcement time, i.e., the reference weights *w*(*k*,*c*), is the set of the normalized amplitudes of the *k*^*th*^ phase (cosine) oscillator. According to (3), the output function of the SBF model with noiseless cosine oscillators is:

(4) $$\;\text{output}(t)=\;\overset{N_{\text{osc}}}{\underset{k=1}{\sum }}w(k,c)w(k,t)=\;\overset{N_{\text{osc}}}{\underset{k=1}{\sum }}\cos(2\pi f_{k}c)\;\cos(2\pi f_{k}t),$$

which becomes:

(5) $$\begin{array}{l} \text{output}(t)=1/2\text{sin}(\pi N_{\text{osc}}\text{df}(t-c))\text{cos}(\pi (2f_{1}+N_{\text{osc}}\text{df})\\ \;(t-c))/\text{sin}(\mathrm{\pi df}(t-c))\\ \;+1/2\text{sin}(\pi N_{\text{osc}}\text{df}(t+c))\text{cos}(\pi (2f_{1}+N_{\text{osc}}\text{df})\\ \;(t+c))/\text{sin}(\mathrm{\pi df}(t+c)). \end{array}$$

**Figure 3:**
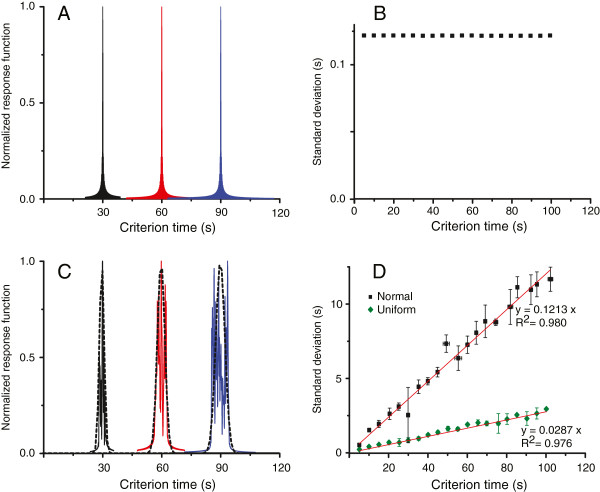
**Normalized output functions for SBF model with cosine oscillators.** In the absence of any variability in the SBF model, the output functions are almost identical regardless the criterion time **(A)** and their widths are constant regardless the criterion times **(B)**. In the presence of uniformly distributed memory variability, the width of the output unction increases with the criterion time **(C)**. The width of the Gaussian envelope (dashed line in **C**) linearly increases with the criterion time both for uniform (solid rhombs in panel **D**) and normally-distributed criterion times (solid squares in panel **D**).

The output function (5) has two symmetric and strong peaks at *t*=±*c*, of which we only retain the first one that has a sharp output when *t*→*c*. We found that 1/2*sin*(*πN*_*osc*_*df*(*t*−*c*))*cos*(*π*(2*f*_1_+*N*_*osc*_*df*)(*t*−*c*))/*sin*(*πdf*(*t*−*c*)) approaches 1/2*N*_*osc*_ as *t*→*c*, which is in agreement with numerical simulations carried out for a network of 1000 noiseless phase oscillators (see Figure 3). Figure 3A shows the numerically generated output function of the SBF model with noiseless phase oscillators for three memorized criteria, i.e., 30s, 60s, and 90s. Our numerical simulations show that the response of the SBF model peaks when the pattern of input activity *w*(*k*,*t*) “lines-up” or “coincides” with the one stored at the reinforcement time, *c*, i.e., this SBF model is able to produce *precise* interval timing. The shape of the output function at each criterion time (see Figure 3A) is captured by the *sinc* function *envelope*(*t*)=*sin*(*πN*_*osc*_*df*(*t*−*c*))/*sin*(*πdf*(*t*−*c*)), which peaks at *t*=*c* (see Eq. (5)). The width of the *sinc* envelope above is the solution of the equation *envelope*(*c*−*width*/2)=1/2*envelope*(*c*), i.e, *sin*(*πN*_*osc*_*dfwidth*/2)/*sin*(*πdfwidth*/2)=1/2*N*_*osc*_, which shows that the *width* of the envelope is independent of the criterion time, i.e., it violates *time-scale invariance* property of interval timing. We conducted numerical simulations with various to-be-timed criteria (see Figure 3B) and found that the width of the output is indeed constant rather than increasing proportionally to the criterion time as required by time-scale invariance property of interval timing. In conclusion, a noiseless SBF model is *accurate* (peaks at criterion time as seen in Figure 3A), but does not exhibit *time-scale invariance* (the width of the output does not scale-up with the criterion time as seen in Figure 3B).

#### Cosine oscillators with arbitrary memory variability

As mentioned in the Introduction, biological noise is ubiquitous both as channel noise affecting the dynamics of individual oscillators [55,56,61-65] and as stochastic synaptic inputs or network background activity [57-60,66,67]. In this implementation of the SBF model, we investigated the effect of criterion time (memory) variability due to noisy storage and retrieval of the criterion time on scalar timing. We found that regardless the *pdf*s of the stochastic variables involved, the output function is (a) always Gaussian and (b) obeys time-scale invariance property. To prove analytically our conjectures, let us assume only criterion time variability and rewrite the output function (4) as follows:

(6) $$\begin{array}{l} \text{output}(t)=1/2\overset{N_{c}}{\underset{j=1}{\sum }}\overset{N_{\text{osc}}}{\underset{k=1}{\sum }}(\cos(2\pi f_{k}(t-c_{j}))\\ \;+\cos(2\pi f_{k}(t+c_{j}))). \end{array}$$

We only considered the physically realizable first term centered at *t*=+*c*, which lead to:

(7) $$\begin{array}{l} \text{output}(t)=1/2\overset{N_{c}}{\underset{j=1}{\sum }}\overset{N_{\text{osc}}}{\underset{k=1}{\sum }}\cos(2\pi f_{k}(t-c_{j}))\\ \;=\overset{N_{c}}{\underset{j=1}{\sum }}\frac{\sin(a_{j}(f_{2}-f_{1}))\cos(a_{j}(f_{2}+f_{1}))}{2\sin(a_{j}\text{df})}, \end{array}$$

where *a*_*j*_=*π*(*t*−*c*_*j*_). In the presence of memory variability, the criterion time is a stochastic variable *c*_*j*_=*c*(1+*x*_*j*_) where *x* has a *pdfp*_*X*_(*x*). Using *pdf* transformation rules [92,93], we found the *pdf* of the new stochastic variable in (7):

(8) $$\;z\;=\;h(x)\;=\;\frac{\sin(\pi (f_{2}\;-\;f_{1})(t-c-\text{cx}))\cos(\pi (f_{2}+f_{1})(t-c\;-\;\text{cx}))}{\sin(\mathrm{\pi df}(t-c-\text{cx}))}\;.$$

The *pdf*_*z*_ of the new stochastic variable *z*(*x*) is related to the *pdf*_*x*_ of the criterion time *p*_*X*_(*x*) through well-known relationship [92,93]: *p*_*Z*_(*z*)=*p*_*X*_(*h*−1(*z*))|*dx*/*dz*|. Based on the central limit theorem, the output function (7), which is a sum over *N*_*c*_ stochastic variables with the *pdf*_*Z*_(*z*), is always a Gaussian regardless the *pdf* of the criterion time.

What about the time-scale invariance property? Is this feature of the output function still preserved regardless the *pdf* of the criterion time? Based on (8), we estimated the time-dependent output function by averaging over the criterion time fluctuations:

(9) $$\;\begin{array}{l} \text{output}(t)\;=\;\;\;\overset{x_{\text{max}}}{\underset{x_{\text{min}}}{\int }}\;\;\frac{\sin(\pi (f_{2}\;-\;f_{1})(t\;-\;c\;-\;\text{cx}))\cos(\pi (f_{2}+f_{1})(t-c-\text{cx}))}{\sin(\mathrm{\pi df}(t-c-\text{cx}))}p_{X}(x)\text{dx}, \end{array}$$

where the range (*x*_*min*_,*x*_*max*_) depends on the type of *pdf*_*X*_(*x*). Based on the first mean theorem for integrals, there exists a value *x*_*min*_<*θ*<*x*_*max*_ such that (9) becomes:

(10) $$\begin{array}{l} \text{output}(t)=(x_{\text{max}}-x_{\text{min}})\\ \;\times \;\frac{\sin(\pi (f_{2}-f_{1})(t-c-\mathrm{c\theta}))\cos(\pi (f_{2}+f_{1})(t-c-\mathrm{c\theta}))}{\sin(\mathrm{\pi df}(t-c-\mathrm{c\theta}))}p_{X}(\theta ). \end{array}$$

To compute the width of the output function we introduced the dimensionless variable *y*=(*t*−*c*)/*cθ*. The width is the value *y*_0_=(*t*_0_−*c*)/*cθ* at which the amplitude of the output function (10) is half its maximum value:

(11) $$\text{output}(y_{0})=1/2\text{output}(0).$$

Using *t*_0_=*c*+*σ*/2, where *σ* is the half-width of the output function (10), the equation (11) becomes:

(12) $$\begin{array}{l} \frac{\sin(\pi (f_{2}-f_{1})\mathrm{c\theta}(1-y_{0}))\cos(\pi (f_{2}+f_{1})\mathrm{c\theta}(1-y_{0}))}{\sin(\mathrm{\pi dfc\theta}(1-y_{0}))}\\ =\frac{\sin(\pi (f_{2}-f_{1})\mathrm{c\theta})\cos(\pi (f_{2}+f_{1})\mathrm{c\theta})}{2\sin(\mathrm{\pi dfc\theta})}, \end{array}$$

and *y*_0_=*σ*/(2*cθ*) with *θ*≠0. If a solution *y*_0_ exists for Eq. (12), then the width *σ* of the output function must obey the scalar property because *σ*=2*cθy*_0_ increases linearly with the criterion time *c*.

We carried out numerical simulations using cosine model with *N*_*c*_ different criterion times distributed around *c*. Figure 3C shows the output of the SBF model when *N*_*c*_ criteria are drawn from a uniform distribution centered on *c*. For the particular realization of the criteria with uniform distribution, it results form Figure 3C that the width of the Gaussian envelope (dashed line) increases with *c*. Figure 3D shows that the width of the Gaussian fit scales linearly with the criterion time both for uniformly distributed criteria (solid rhombs in Figure 3D) and normal distribution (solid square in Figure 3D). These results support our theoretical prediction that the scalar property is valid regardless the *pdf* of memory variability.

### The SBF with biophysically realistic model oscillators

Cosine oscillators were extensively used in numerical simulations of interval timing models with great success [13,21,52]. Our current theoretical predictions and numerical simulations of SBF model with cosine oscillators are in good agreement with interval timing experiments. Cosine model has a series of advantages: (1) it is mathematically convenient and computationally efficient, (2) it is close to actual voltage traces recorded from neural oscillators that fire close to a critical (bifurcation) point, and (3) it helps us understand the effects of different types of variabilities (noises) on the output of the SBF interval timing model. However, the cosine waveforms are not physiologically realistic. Furthermore, abstract cosine waveforms, cos(2*πft*), cannot be linked with the biophysics involved in action potential firing, such as the density of ionic channels, membrane capacitance, etc. Another consequence of these shortcomings is that the cosine waveform cannot account for the effect of neuromodulators since there is no biophysical mechanism behind the cosine oscillators with fixed frequency. Therefore, as highlighted in the Introduction, we replaced the cosine oscillator with a ML model neuron [68,69] for two main reasons (see Appendix for model equations): 1) ML model neuron is one of the simplest and often used as a realistic cortical oscillator model [74,94] that includes conductance-based mechanisms similar to Hodgkin-Huxley model [70] involving potassium and calcium channels and, 2) by changing a relatively small subset of model’s parameters, ML model neuron can act as a Type 1 excitable cell (fast spiking) [73] or a Type 2 excitable cell (slowly sinusoidal envelope close to a cosine waveform) [95].

#### ML oscillators with no variability

In the absence of any variability, our numerical results show that the width of the output function of the SBF model with ML oscillators does not change with criterion time, therefore, violating the scaling property. This finding is not surprising and it was predicted analytically in the case of cosine models. Since any periodic waveform, such as the action potential of an endogenously spiking neuron, can be decomposed in discrete cosine components, we conjectured that “no variability = no scalar property” based on the theoretical results obtained with cosine oscillators. We also noticed that the width of the output function decreases with the increase in the number of neural oscillators. Based on our cosine oscillator results, this observation is also predicable since the output function is the discrete Fourier transform of the reference weights vector *w*(*k*,*c*). Since the temporal and frequency domains are conjugated through a Fourier transform [96], the product *ΔfΔt* is constant. Therefore, increasing the frequency resolution *Δf* (by increasing the number of neural oscillators recruited for interval timing tasks over the same frequency range) decreases the temporal spread of the output function and makes the behavioral response more localized.

#### ML oscillators with arbitrary memory variability

The fact that noise, whether as channel noise [61-63] or background synaptic activity [59,60] is a crucial ingredient that often leads to new and unexpected effects is not limited to interval timing. For example, the noise facilitates signal transduction [97] and enhances signal detection by sensory systems [98]. The noise also induces synchronization of neural oscillations in olfactory bulb mitral cells [99] or in large networks cortical fast-spiking cells [100]. Cortical neurons have a large coefficient of variation of the interspike interval [64,65] which can be modeled at different levels of details from an explicit ML stochastic models of ionic channels to phenomenological potential-dependent averages [101]. In this paper, we opted for a phenomenological approach to modeling variability in the interspike interval through a fluctuating bias current.

**Figure 4:**
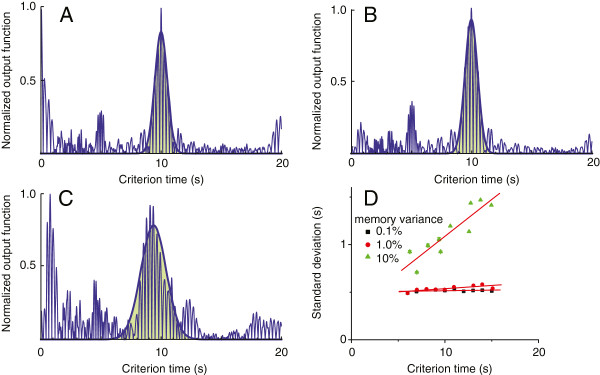
**Normalized output functions for SBF model with ML oscillators.** The width of the output function of the SBF model with 600 ML model neurons in the frequency range from 5.5 Hz to 11.5 Hz with normally distributed criterion times is insensitive to low 0.1*%***(A)** and 1% **(B)** levels of noise. The width of the output function shows linear increase with the criterion time for high noise levels, i.e., 10% **(C)** and panel **D**.

In order to maintain the parallel with the cosine (phase) model, we report here only the effect of memory variability on the standard deviation of the Gaussian fit of the output function generated by the SBF model with ML oscillators (see Figure 4). First, we noticed from our numerical simulations that the SBF model with ML neurons is less sensitive to the level of memory noise. For example, a noise level of 0.1*%* that led to a linear dependence of the standard deviation on the criterion time in the case of the SBF model with cosine oscillators produced no significant change in the width of the output function with ML neurons (Figure 4D). The scalar property is indeed valid (Figure 4D), but it emerges at higher levels of memory variability, which were not even accessible to phase model. The slope of the standard deviation was insignificant 0.001±0.001(*R*^2^=0.342) for 0.1*%* memory variance (Figure 4A), 0.007±0.002(*R*^2^=0.789) for 1% variance (Figure 4B), respectively, 0.07±0.01(*R*^2^=0.898) for 10% memory variance (Figure 4C). We found that a ten fold increase in memory variability (from 1% to 10%) led to a ten fold increase (from 0.007 to 0.07) in the slope of the standard deviation versus criterion time. This result suggests that for the SBF model with ML oscillators *σ*_*output*_∝*σ*_*c*_*c* as we predicted and already checked for the SBF model with phase oscillators.

## Discussion

Interval timing models vary largely with respect to the fundamental assumptions and the hypothesized mechanisms by which temporal processing is explained. In addition, interval timing model attempt explaining time-scale invariance, or drug effects differently. Among the most prominent models of interval timing we cite pacemaker/accumulator processes [4-6], sequences of behaviors [102], pure sine oscillators [13,16,21,45], memory traces [103-107], or cell and network-level models [108,109]. Both neurometric functions from single neurons and ensemble of neurons successfully paralleled the psychometric functions for the to-be-timed intervals shorter than one second [108]. Interacting populations that balance LTP and LTD mechanisms are thought to modulate the firing rate of single-cell with the end effect at the population level that the adaptation leads to a linear decay of the firing rate over time [110]. Therefore, the linear relationship between time and the number of clock ticks of the pacemaker-accumulator model in the SET of interval timing [4] was translated into a linearly decaying firing rate model that maps time and variable firing rate.

By and large, to address time-scale invariance current behavioral theories assume convenient computations, rules, or coding schemes. Scalar timing is explained as either deriving from computation of ratios of durations [4]*-*[6,111], adaptation of the speed at which perceived time flows [102], or from processes and distributions that conveniently scale-up in time [45,103,105,106]. Some neurobiological models share computational assumptions with behavioral models and continue to address time-scale invariance by specific computations or embedded linear relationships [112]. Some assume that timing involves neural integrators capable of linearly ramping up their firing rate in time [109], while others assume LTP/LTD processes whose balance leads to a linear decay of the firing rate in time [110]. It is unclear whether such models can account for time-scale invariance in a large range of behavioral or neurophysiological manipulations.

For example, Killeen and Taylor (1988) explained time-scale invariance of timing in terms of noisy information transfer during counting. Similarly, here, we explained time-scale invariance of timing in terms of noisy coincidence detection during timing. Our theoretical predictions based on an SBF model show that time-scale invariance emerges as the property of a (very) large and noisy network. Furthermore, our results regarding the effect of noise on interval timing support and extend the speculation [21] by which an SBF model requires at least one source of variance (noise) to address time-scale invariance. Rather than being a signature of higher-order cognitive processes or specific neural computations related to timing, time-scale invariance naturally emerges in a massively-connected brain from the intrinsic noise of neurons and circuits [1,108]. This provides the simplest explanation for the ubiquity of scale invariance of interval timing in a large range of behavioral, lesion, and pharmacological manipulations.

## Conclusions

We investigated both analytically and numerically the properties of the output function generated by the SBF model and found that the output function mimics behavioral responses of animals performing peak interval procedures. We found analytically that, in the absence of any kind of variability in the parameters of the SBF model, the width of the output function only depends on the number of oscillators and the range of frequencies they cover. Therefore, in the absence of parameter variability the scalar property is violated.

We showed that if parameter variability is allowed, then the output function of the SBF model with cosine oscillators is always Gaussian, which is a consequence of the central limit theorem, regardless the *pdf* of the fluctuating parameter(s). Moreover, we found that the scalar property is also preserved regardless the *pdf* of the fluctuating parameter(s).

We also conjectured that the following two statements are always true in any noisy SBF implementation: (1) the output function is always Gaussian, which is a consequence of central limit theorem, and (2) the scalar property is valid regardless the *pdf* of the fluctuating parameter(s). The justification for such general statements is that any periodic waveform of an endogenously spiking neuron can be decomposed into a sum of cosine waves. Based on our theoretical proof that any SBF model with noisy cosine oscillators has a Gaussian output function that obeys the scalar property, we concluded that the biophysical details of the oscillators that generate the train of periodic action potentials are not relevant for the shape of the output function or the validity of the scalar property. Our numerical tests of the SBF model with biophysically realistic periodically spiking ML model neurons showed that the above two conjectures are valid.

## Appendix

### Cosine model with no variances violates the scalar property

Close to the criterion time, c, only the fist term in (5) is significant. We used the least square fit method to approximate its envelope with a Gaussian centered on the criterion time. The output function becomes:

(13) $$\text{output}(t)=1/2\text{sin}(N_{\text{osc}}x)\text{cos}((2f_{1}/\text{df}+N_{\text{osc}})x)/\text{sin}(x),$$

where *x*=*πdf*(*t*−*c*). The envelope of the output function (13) is given by the maxima of *sin*(*N*_*osc*_*x*)/*sin*(*x*), which oscillates much slowly than the *cos*((2*f*_1_/*df*+*N*_*osc*_)*x*) factor. Therefore, the local maxima of the absolute value of the output function (13) are determined by the zeroes of the first derivative of *sin*(*N*_*osc*_*x*)/*sin*(*x*), i.e., solutions of *tan*(*N*_*osc*_*x*_0_)=*N*_*osc*_*tan*(*x*_0_). The corresponding maximum values of the output function (13) are:

(14) $$y_{0}=1/2\text{sin}(N_{\text{osc}}x_{0})\text{cos}((2f_{1}/\text{df}+N_{\text{osc}})x_{0})/\text{sin}(x_{0}).$$

The pairs (*x*_0_,*y*_0_) are determined by the number of oscillators *N*_*osc*_ in the network and the range of frequencies covered. However, since there is no dependence of (*x*_0_,*y*_0_) pair on the criterion time the output function is simply centered on *t*=*c* but otherwise totally independent on *c*. This means that the width *σ*_*out*_ of the output function envelope depends only on the range of oscillators’ frequencies *f*_1_ and *f*_2_=*N*_*osc*_*df* and the number of oscillators, but is independent of the criterion time, therefore, violating the scalar property.

### Morris-Lecar model equations

We used a dimensionless, conductance-based, Morris-Lecar model [68,113] described by the following equations:

(15) $$\begin{array}{ll} x_{1}^{\prime }= & \;f_{1}(x_{1},x_{2})=-\;I_{\text{Ca}}-I_{K}-I_{L}+I_{0},\\ x_{2}^{\prime }= & \;f_{2}(x_{1},x_{2})=\xi \lambda_{0}(x_{1})(w_{\infty }(x_{1})-x_{2}), \end{array}$$

where *x*_1_ is the membrane potential, *x*_2_ is the slow potassium activation and all ionic currents are described by *I*_*x*_=*g*_*x*_(*x*_1_−*E*_*x*_), where *g*_*x*_ is the conductance of the voltage gated channel *x* and *E*_*x*_ is the corresponding reversal potential. In particular, the calcium current is *I*_*Ca*_=*g*_*Ca*_*m*_*∞*_(*x*_1_)(*x*_1_−*E*_*Ca*_), the potassium current is *I*_*K*_=*g*_*K*_*x*_2_(*x*_1_−*E*_*K*_), and the leak current is *I*_*L*_=*g*_*L*_(*x*_1_−*V*_*L*_). The reversal potentials for calcium, potassium and leak currents are *E*_*Ca*_=1.0,*E*_*K*_=−0.7,*E*_*L*_=−0.5, respectively. The steady state activation function for calcium channels is *m*_*∞*_(*x*_1_)=1+ tanh((*x*_1_−*V*_1_)/*V*_2_))/2, where *V*_1_=−0.01,*V*_2_=0.15, the steady state activation function for potassium channels is *w*_*∞*_(*x*_1_)=(1+ tanh((*x*_1_−*V*_3_)/*V*_4_])/2 where *V*_3_=0.1,*V*_4_=0.145, the inverse time constant of potassium channels is *λ*_0_(*x*_1_)= cosh((*x*_1_−*V*_3_)/*V*_4_/2), the potassium and leak conductances are *g*_*K*_=2.0,*g*_*L*_=0.5, respectively, and the *ξ*=1.0/3.0.

The two control parameters that can switch the ML model from a Type 1 excitable cell [70] to a Type 2 are the calcium conductance *g*_*Ca*_ and the bias current *I*_0_. If *g*_*Ca*_=1.0 and 0.083<*I*_0_<0.242 the equations (15) describe what was classified by A.L. Hodgkin as Type 1 excitable cells. If *g*_*Ca*_=0.5 and 0.303<*I*_0_<0.138 the equations (15) describe a Type 2 excitable cells. In our simulations, we used a Type 2 ML model neuron that has a membrane potential shape very close to a cosine waveform.

## Competing interests

The authors declare no disclosure of financial interests and potential conflict of interest.

## Authors’ contributions

The analytical results regarding the Gaussian shape of the output function and the scalar property were obtained by SAO. CVB contributed to the implementation of the SBF model with cosine oscillators. SAO implemented the SBF model with ML model neurons and conducted all numerical simulations. Both authors contributed equally to the draft the manuscript. Both authors read and approved the final manuscript.
